# Handbook of the Practice of Medicine

**Published:** 1879-04

**Authors:** 


HANDBOOK OF THE PRACTICE OF MEDICINE. By M. CHARTERIS, M.D., Professor of Practice of Medicine, Anderson's College, Glasgow, etc., with Illustrations. Lindsay & Blakiston, Philadelphia. 1878.

While this is an epitomised delineation of the various physiological, pathological and therapeutical processes usually found in unabridged works on the practice of medicine, it is a goodly sized volume, and constitutes as complete a reference book as could be gotten up. The illustrative diagrams of comparative anatomy and pathalogical specimens are comprehensive and well executed. As a complete compendium of the practice of medicine, we commend it to those who desire ready reference to fresh and authentic information.

